# Metabolomics and Dual RNA-Sequencing on Root Nodules Revealed New Cellular Functions Controlled by *Paraburkholderia phymatum* NifA

**DOI:** 10.3390/metabo11070455

**Published:** 2021-07-15

**Authors:** Paula Bellés-Sancho, Martina Lardi, Yilei Liu, Leo Eberl, Nicola Zamboni, Aurélien Bailly, Gabriella Pessi

**Affiliations:** 1 Department of Plant and Microbial Biology, University of Zürich, CH-8057 Zürich, Switzerland; paula.belles@botinst.uzh.ch (P.B.-S.); lardimartina@gmail.com (M.L.); yilei.liu@botinst.uzh.ch (Y.L.); leberl@botinst.uzh.ch (L.E.); 2 ETH Zürich, Institute of Molecular Systems Biology, CH-8093 Zürich, Switzerland; zamboni@imsb.biol.ethz.ch

**Keywords:** symbiosis, nitrogenase, C_4_-dicarboxylates, auxin, brassinosteroid, flavonoid, stress response

## Abstract

*Paraburkholderia phymatum* STM815 is a nitrogen-fixing endosymbiont that nodulate the agriculturally important *Phaseolus vulgaris* and several other host plants. We previously showed that the nodules induced by a STM815 mutant of the gene encoding the master regulator of nitrogen fixation NifA showed no nitrogenase activity (Fix^−^) and increased in number compared to *P. vulgaris* plants infected with the wild-type strain. To further investigate the role of NifA during symbiosis, nodules from *P. phymatum* wild-type and *nifA* mutants were collected and analyzed by metabolomics and dual RNA-Sequencing, allowing us to investigate both host and symbiont transcriptome. Using this approach, several metabolites’ changes could be assigned to bacterial or plant responses. While the amount of the C_4_-dicarboxylic acid succinate and of several amino acids was lower in Fix^−^ nodules, the level of indole-acetamide (IAM) and brassinosteroids increased. Transcriptome analysis identified *P. phymatum* genes involved in transport of C_4_-dicarboxylic acids, carbon metabolism, auxin metabolism and stress response to be differentially expressed in absence of NifA. Furthermore, *P. vulgaris* genes involved in autoregulation of nodulation (AON) are repressed in nodules in absence of NifA potentially explaining the hypernodulation phenotype of the *nifA* mutant. These results and additional validation experiments suggest that *P. phymatum* STM815 NifA is not only important to control expression of nitrogenase and related enzymes but is also involved in regulating its own auxin production and stress response. Finally, our data indicate that *P. vulgaris* does sanction the *nifA* nodules by depleting the local carbon allocation rather than by mounting a strong systemic immune response to the Fix^−^ rhizobia.

## 1. Introduction

Nitrogen-fixing symbiosis between rhizobia and plants of the Fabaceae family significantly contributes more than half of the biosphere’s available nitrogen to agriculture [1]. This process is known as biological nitrogen fixation (BNF) and takes place in specialized root and stem organs, called nodules, where rhizobia differentiate into bacteroids and convert atmospheric nitrogen into ammonium [2]. This enzymatic conversion, carried out by the oxygen-sensitive bacterial enzyme nitrogenase, requires large amounts of energy and reductants. The plant supplies bacteroids with carbon sources such as the C_4_-dicarboxylates succinate, malate and fumarate, which are imported by the C_4_-dicarboxylate transport (Dct) systems and are utilized as energy source [3,4]. Rhizobia are a taxonomically diverse group of bacteria, which belong to the alpha- and beta-subclass of proteobacteria [5,6]. *Paraburkholderia phymatum* STM815 is an aerobic soil bacterium, which was isolated in 2001 and has since been shown to nodulate the roots of a broad range of mimosoid and papilionoid plants and develop a nitrogen-fixing symbiosis [7,8,9,10,11,12].

The establishment of an effective symbiosis between rhizobia and fabacean plants involves a complex molecular dialogue between the two partners, which is based on different plant and rhizobial molecules. In response to specific flavonoids secreted by the host species, rhizobia induce the expression of the so-called Nod factors (lipochitooligosaccharides), which lead to root hair curling and trapping of bacterial colonies as well as to growth and reprogramming of root cortex cells, finally leading to the formation of a nodule [5]. Commonly through infection threads, rhizobia penetrate the deeper plant tissue and are released intracellularly and surrounded by a symbiosome membrane. Inside plant cells, they differentiate into nitrogen-fixing bacteroids [13,14]. The oxygen concentration inside nodules is roughly 11 nM, which corresponds to a 10,000-fold lower concentration compared to aerobic conditions [14,15]. This oxygen-limited environment is important to protect nitrogenase activity and thereby initiate the expression of nitrogen fixation (*nif*) genes [14,16]. In diazotrophic bacteria and in *P. phymatum*, the sigma factor RpoN (or sigma-54) and its enhancer binding protein NifA have been shown to be essential for activation of *nif* gene expression and nitrogenase activity [17,18,19]. NifA activity is oxygen-sensitive due to the presence of conserved cysteine residues, which sense the oxygen level and allow the protein to assume its active form inside the nodules. The regulon of NifA was determined in several diazotrophs mainly using transcriptome and proteome analysis [20,21,22,23,24,25]. However, so far no study has described the NifA regulon in beta-rhizobia. We previously showed that plants inoculated with a *P. phymatum nifA* mutant were not only deficient in reducing atmospheric nitrogen, but also showed an increased number of nodules and displayed a grape-shaped structure on the roots [18].

Here, we successfully integrate metabolomics with dual RNA-Sequencing of *Phaseolus vulgaris* root nodules infected by a *P. phymatum nifA* mutant or by the wild-type. Using this strategy, we were able to assign metabolomics changes to the bacterium (*P. phymatum*) or to the plant host (*P. vulgaris*) and thereby discovered that *P. phymatum* NifA is not only a master regulator of genes involved in nitrogen fixation but also controls other potentially important symbiotic traits and induces substantial changes in the plant host. Together, this study illustrates the value of combing metabolomics with a dual RNA-Seq approach to tackle the biology of intracellular rhizobium-plant symbiotic interactions. 

## 2. Results and Discussion

### 2.1. Metabolomics Analysis of Nodules Infected by a nifA Mutant Strain

**Figure 1:**
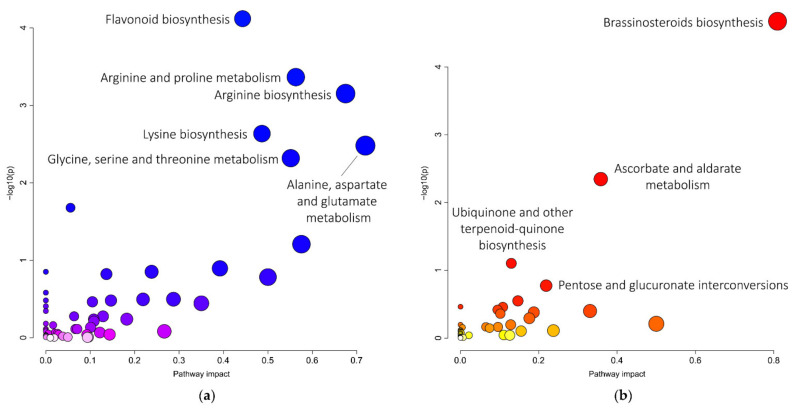
KEGG metabolic pathway enrichment and pathway topology analysis. Over-represented KEGG metabolic pathways in bean nodules colonized by *P. phymatum* wild-type compared to nodules colonized by *P. phymatum nifA* mutant (**a**) and over-represented in *nifA* compared to wild-type nodules (**b**). The metabolites used for this representation display different levels in both types of nodules (absolute log_2_FC < 0.5 and *q*-values ≤ 0.01).

**Figure 2:**
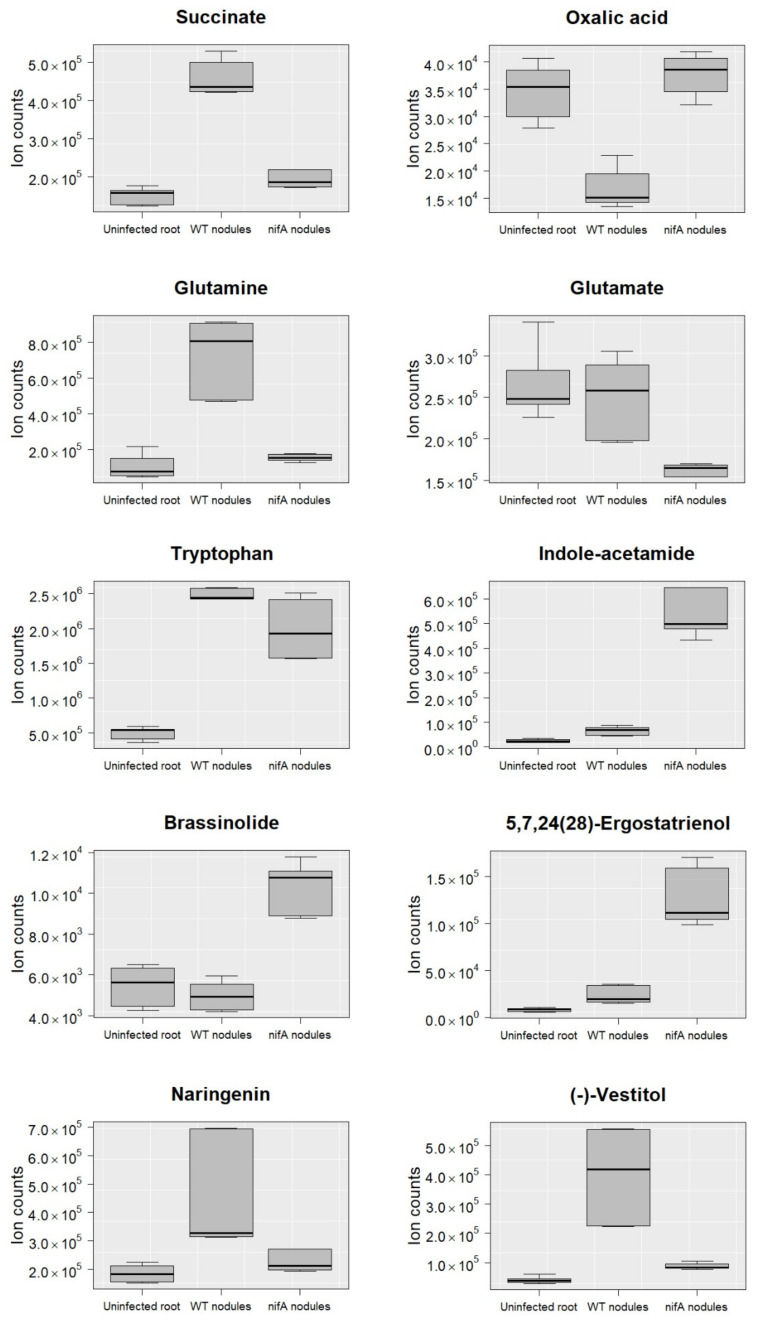
Comparison of the ion counts detected for each metabolite in uninfected bean roots (Uninfected root), nodules infected with *P. phymatum* wild-type (WT nodules) and nodules infected with *P. phymatum nifA* mutant (nifA nodules). The following compounds were examined: succinate, oxalic acid, glutamine, glutamate, tryptophan, indole-acetamide, brassinolide, 5,7,24(28)-ergostatrienol, naringenin and (−)-vestitol. Three biological replicates with two technical replicates were analyzed and are shown as boxplots (median in bold).

In order to compare the metabolic status of the nodules occupied by either wild-type and *nifA* mutant strain, a non-targeted metabolomics analysis was performed on common bean nodules (21 days post infection) as described in the material and methods section. Overall, 526 metabolites could be identified, of which 172 showed differential levels in the *nifA* mutant versus wild-type nodules (Supplementary Table S1). Most of these (107) showed a decreased amount in nodules formed by the *nifA* mutant and approximately a fourth of them (24) were previously shown to be specifically accumulating in nodules formed by *P. phymatum* wild-type compared to uninoculated roots [26]. The absence of NifA in nodules was associated with a reduction of the content of the amino acids alanine, glycine, proline and tyrosine, threonine, glutamine, asparagine, lysine, glutamate and ornithine (Figure 1a). Several amino acid precursors showed also lowered levels in nodules infected by the *nifA* mutant, which suggests a reduced assimilation of nitrogen: 3-dehydroquinate and chorismate (tyrosine bioynthesis), phosphohomoserine (synthesis of threonine and glycine), aminoadipate and homocitrate (lysine biosynthesis), l-glutamyl 5-phosphate (synthesis of proline) and oxoglutarate, N-acetyl-glutamate and N-acetyl-ornithine. Aspartate was the only amino acid that was increased in nodules occupied by the *nifA* mutant. Moreover, the level of phosphoaspartate also increased in Fix^−^ nodules. For the tryptophan metabolic pathway, we observed an accumulation of formyl-anthranilate and a decreased amount of anthranilate. *Sinorhizobium meliloti* Rmd201 anthranilate synthase (*trpE*) mutants have been shown to be ineffective in nitrogenase activity inside alfalfa root nodules [27,28]. The one-carbon and purine biosynthesis intermediate 10-formyl-tetrahydrofolate was less abundant in nodules occupied by the *nifA* mutant. The level of the C_4_-dicarboxylate succinate decreased significantly in the non-fixing nodules containing the *nifA* mutant compared to wild-type nodules, reaching similar levels to uninfected roots (Figure 2). Succinate together with arginine (which also showed decreased levels in *nifA* nodules) is one of the major carbon supplies that the host plant offers to the bacteroids in exchange for ammonia [29]. Therefore, the plant may sanction the Fix^−^ bacteroids by interrupting carbon supply. On the other hand, the level of other compounds involved in glyoxylate and dicarboxylate metabolism, glycolate, oxalate and hydroxypyruvate as well as the important intermediate in carbon metabolism phospho*enol*pyruvate (PEP) was higher in nodules infected by the *nifA* mutant relative to wild-type nodules. Interestingly, the higher oxalate level in nodules occupied by the *nifA* mutant (Figure 2) may indicates that—similarly to *Bradyrhizobium efficiens* [30]—*P. phymatum* is able to use oxalate as carbon and energy source. Indeed, a genomic inspection in *P. phymatum* genome revealed the presence of the key pathway genes *oxc* (for oxalyl-coenzyme A decarboxylase) and *frc* (for formyl-coenzyme A transferase). However, these genes were not differentially expressed in wild-type and mutant nodules (see below). Two products of nitrogen fixation known to be assimilated in determinate common bean nodules, allantoin and allantoate, as well as their precursors hydroxyisourate and the purine guanine showed reduced levels in nodules infected by the *nifA* mutant. GTP and the allantoate precursor inosine showed the opposite trend, i.e., accumulation in non-fixing nodules. 

Among the metabolites showing decreased levels in nodules infected by the *nifA* mutant we found catechol and cyclohexanone, which are intermediates in the degradation of aromatic compounds. Several flavonoids and isoflavonoids were also found among the metabolites less accumulated in *nifA* mutant nodules (putatively assigned to naringenin, luteolin, (−)-vestitone, (−)-vestitol, 3,9-dihydroxypterocarpan, biochanin A) (Figure 1a). Since flavonoids are important signals used by the plants to communicate with their rhizobial partners [31,32], we speculate that in nodules occupied by the *nifA* mutant, the communication between bacteria and plant may be impaired. Moreover, flavonoids are recognized auxin transport inhibitors, which could affect plant growth [33]. The level of flavonoids such as naringenin and (−)-vestitol in nodules occupied by the *nifA* mutant was reduced to similar levels detected in uninfected roots (Figure 2). In contrast, several compounds involved in steroid and brassinosteroid biosynthesis were among the 65 compounds accumulating in bean nodules occupied by the *nifA* mutant (Figure 1b). Brassinosteroids (BR) are plant hormones, which promote cell division [34] and inhibit infection thread formation [35]. They have been shown to interact with auxin leading to its accumulation in the cortical tissue [36]. Indeed, the most highly accumulated metabolite in nodules occupied by the *nifA* mutant was the auxin indole-acetamide (IAM) (Supplementary Table S1, Figure 2). Another auxin, the well-investigated indole-3-acetic acid (IAA) was found to be 1.4-fold increased, although the difference with wild-type nodules was not considered as significant with the thresholds we applied (log_2_ fold change ≥0.5 and *q*-value ≤ 0.01). Auxins play multiple roles in plants and accumulate in infected root hairs, facilitating infection-thread formation during *Mesorhizobium loti*–*Lotus japonicus* symbiosis [37]. The increase in auxins is in agreement with a slight decrease in tryptophan (Figure 2), the precursor of auxins, suggesting that the observed accumulation of auxins occurred via the tryptophan-dependent pathway. Without further microscopic investigations, it is premature to interpret the impact of the imbalanced BR/auxin homeostasis and flavonoids contents on the organogenesis and development of *nifA*-colonized nodules. In contrast to indeterminate nodules, hormonal cross-talks and flavonoids regulation are less described in determinate nodules. However, genetic work in *M. truncatula* suggested BR as AON-independent promoters of nodule number, and the role of auxins in specifying nodule founder cells and the progression of nodule growth is well-established [38,39].

### 2.2. RNA-Sequencing and P. phymatum Differential Gene Expression

After mapping the reads to *P. phymatum* genome, we observed 479 bacterial genes to be differentially expressed (Supplementary Table S2, see Material and Methods, Section 3.4) in 21 days post infection (dpi) nodules infected by the wild-type or the *nifA* mutant strain. Out of the 479 rhizobial genes, 142 (roughly 30%) were located on the symbiotic plasmid pBPHY02, confirming the importance of this transcriptional regulator during symbiosis (Table 1). 

**Figure 3:**
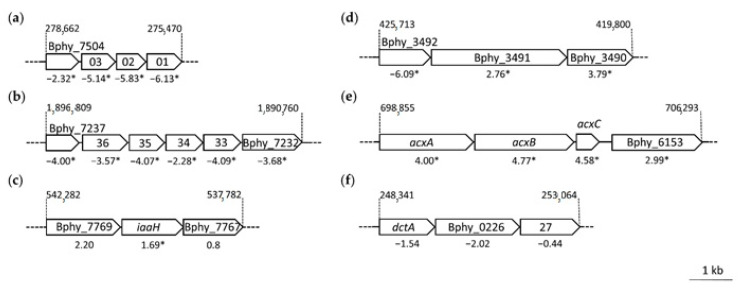
A selection of *P. phymatum* operons showing differential expression in *nifA* nodules compared to wild-type nodules. The following operons are represented: (**a**) lysine/arginine/ornithine ABC transporter; (**b**) alkane sulfonate ABC transporter; (**c**) indole-3-acetic acid synthesis (*iaa*); (**d**) efflux RND transporter; (**e**) acetone carboxylase (*acx*); (**f**) dicarboxylate symporter (*dct*). The genomic coordinates according to the replicon where the operon belongs are indicated at the beginning of the operon and gene names are indicated in italic. The log_2_FC values are shown underneath the operons, with statistically significant ones marked with an asterisk.

Among the 367 genes showing down-regulation in the *nifA* mutant, we found several *nif* and *hyp* genes, coding for nitrogenase and hydrogenase-related genes. Consistent with a decrease in homocitrate levels, the expression of the *P. phymatum* homocitrate synthase encoding gene *nifV* (Bphy_7741) was down-regulated in nodules in absence of NifA. We recently showed that NifV is crucial for nitrogenase function during free-living growth and during symbiosis with papilionoid plants [26]. Moreover, the cytochrome o ubiquinol oxidase (Bphy_3649-46, *cyoABCD*), which we previously identified to be important for *P. phymatum* symbiosis with common bean, was also among the top genes positively regulated by NifA [18]. In line with the decrease in glutamine observed in our metabolomics analysis (Figure 2), we found Bphy_6981 coding for a glutamine synthetase to be down-regulated in *nifA* mutant bacteroids (Supplementary Figure S1a). Furthermore, Bphy_5298 and Bphy_7471, coding for a glutamine amidotransferase class I and class II, respectively, showed decreased expression in *nifA* bacteroids. Glutamine amidotransferases are involved in nitrogen assimilation and generate ammonia by glutamine hydrolysis, which is then transferred to a specific substrate. Congruent with the down-regulation of the pyridoxal-phosphate dependent enzyme encoded by Bphy_7498 that converts *o*-phospho-L homoserine into threonine, we found a decrease of both compounds in nodules occupied by a *nifA* mutant. The decreased expression of Bphy_5639 encoding an aspartate ammonia-lyase (AspA) that converts aspartate into the C_4_-dicarboxylate fumarate, may explain the accumulation of aspartate in nodules infected by the *nifA* mutant (Supplementary Figure S1a). Moreover, the decrease of L-aspartate-4-semialdehyde and 2,4 diaminobutanoate could be connected to the down-regulation of the Bphy_7266 (*ectB*) transcript coding for a diaminobutyrate-2-oxoglutarate transaminase. The operon Bphy_7504-01 coding for a potential lysine/arginine/ornithine ABC transporter located on the symbiotic plasmid was down-regulated and reflected the decrease of the metabolites lysine and ornithine in *nifA* bacteroids (Figure 3a). Another operon (Bphy_7232-37) located on pBPHY01 encoding a potential alkane sulfonate ABC transporter showed less expression in a *nifA* mutant compared to the wild-type during symbiosis (Figure 3b). An ABC transporter-like protein for transport of branched amino acids (Bphy_3041) was down-regulated at the transcript level. The two-component regulatory system NarXL (Bphy_6191/6192) as well as the two nitrate reductase genes *narG* and *narH* showed decreased expression in nodules occupied by the *nifA* mutant, suggesting that NifA is involved in the control of nitrate metabolism in bacteroids. In addition to the previously mentioned alkane sulfonate ABC transporter, expression of several other genes involved in sulfur metabolism was down-regulated in the *nifA* mutant compared to wild-type bacteroids. For example, the sulfite dehydrogenase complex SoxCD (Bphy_7230-31), a potential taurine transporter (Bphy_6080/81), the sulfurtransferase Bphy_6245 converting thiosulfate into sulfite, Bphy_2231 coding for a sulfate adenylyltransferase, Bphy_3604 encoding a TauD/TfdA family dioxygenase, which potentially converts taurine into sulfite, and Bphy_7479 potentially involved in producing cysteine from sulfide. Additionally, *cysN* (Bphy_2231), coding for a sulfate adenylyltransferase was also down-regulated in bacteroids missing *nifA*. The expression of *treS* (Bphy_7407) coding for a trehalose synthase was decreased in *nifA* mutant bacteroids compared to wild-type bacteroids. However, our metabolomics data showed the amount of trehalose did not change in *nifA* nodules compared to wild-type nodules. Among the down-regulated genes we also found one potential ferritin (Bphy_1509) that are known to confer protection against oxidative stress by oxidizing ferrous ions (Fe^2+^) to ferric ions (Fe^3+^) [40].

Among the 112 *P. phymatum* genes with up-regulated expression in *nifA* mutant bacteroids, only five were located on the symbiotic plasmid: Bphy_7724 and Bphy_7726 coding for a putative biotin carboxylase and a hypothetical protein (HP) with a heme oxygenase domain, respectively; Bphy_7816 (HP), Bphy_7384 encoding a histone family protein DNA-binding protein and Bphy_7768 (*iaaH*) coding for an indole acetamide (IAM) hydrolase (Figure 3c) (Supplementary Figure S1b). The up-regulation of *P. phymatum iaaH* expression strongly suggests that the accumulated auxin in nodules infected with the *nifA* mutant is synthetized by the bacteria. Several genes involved in the response to stress showed increased expression in non-fixing bacteroids such as Bphy_2521 (catalase) and Bphy_3656 (alkyl hydroperoxide reductase subunit F), both involved in the reaction to oxidative stress, a protease with a HtrA/DegQ protease family signature (Bphy_0471) and Bphy_0301 coding for the RpoH sigma factor. RpoH has been discovered as the main regulator of the heat-shock response in *E. coli* thereby regulating transcription of genes coding for chaperones and proteases [41]. In addition to heat response, RpoH has been shown to play a role in oxidative stress response, for osmotic tolerance, virulence and during nitrogen-fixing symbiosis [42,43]. In contrast to alpha-rhizobial strains, which usually display two or three copies of *rpoH* in their genome [44], *P. phymatum* possesses only this single copy. A *Sinorhizobium meliloti rpoH_1_* mutant was able to nodulate alfalfa plants but failed to fix nitrogen inside nodules. Both *S. meliloti rpoH_1_* and *rpoH_2_* copies were shown to be involved in the regulation of the expression of heat shock proteins. Curiously, *P. phymatum* RpoH showed higher homologies to *S. meliloti rpoH_1_* (42% identities, 61% similarities at the amino acid level). The up-regulation of transcripts involved in the stress response suggests that *nifA* mutant bacteroids are stressed inside the nodule. Among the statistically significant up-regulated genes, several were organized in a predicted operon structure such as Bphy_3490-92 coding for an efflux RND transporter (Figure 3d), Bphy_4949-50 probably involved in fatty acid metabolism, Bphy_5790-92 containing a beta-lactamase domain-containing protein and a reductase, Bphy_6150-52 encoding a potential acetone carboxylase that catalyzes the condensation of acetone and CO_2_ to form acetoacetate in order to grow with acetone as carbon source and electron donor for respiration (Figure 3e). The *acxABC* operon in *Helicobacter pylori* has been shown to be important for colonization of the gastric mucosa and may therefore play a role in survival of the bacterium in the stomach [45]. Remarkably, the expression of the sigma-54-dependent transcriptional regulatory gene Bphy_6153 located downstream of this operon was also increased in nodules occupied by the *nifA* mutant, suggesting that Bphy_6153 controls acetone carboxylase expression (Figure 3e). Moreover, two genes coding for two enzymes involved in the TCA cycle showed higher expression in *nifA* nodules compared to wild-type nodules: Bphy_5588 encoding a succinate dehydrogenase flavoprotein subunit potentially converting succinate into fumarate and Bphy_4692 coding for a succinyl-CoA synthetase (Supplementary Figure S1a). Our metabolomics data showed that succinate and 2-oxoglutarate were less abundant in non-fixing nodules formed by the *nifA* mutant compared to wild-type nodules, suggesting that *nifA* mutant bacteroids may try to provide itself energy and carbon sources.

### 2.3. RNA-Sequencing and P. vulgaris Differential Gene Expression

Out of the 1572 *P. vulgaris* differentially expressed genes (see Material and Methods, Section 3.4), 337 transcripts were found more abundant in nodules colonized by the *nifA* mutant (Supplementary Table S3). Apart from few putative defense-related genes, counting three diseases resistance-responsive (dirigent-like protein) family proteins, five protein kinases and three ABCG transporters, *nifA* nodules do not present a strong immune response (Supplementary Table S4). A notable exception to this trend was the strong overexpression of the *PATHOGENESIS-RELATED* protein 1 (PR-1) family member PHAVU_006G196900g. PR-1 proteins represent common markers of the late, salicylic acid-driven pathogenic response [46]. Similarly, besides a WRKY transcription factor (PHAVU_004G105800g), a protein belonging to the *ENHANCED DISEASE SUSCEPTIBILITY TO ERYSIPHE ORONTII* family (PHAVU_010G011700g) and three disease resistance proteins (PHAVU_010G028700g, PHAVU_010G027300g and PHAVU_010G028800g), very few genes related to this functional category were found significantly down-regulated in *nifA* nodules compared to *P. phymatum* wild-type nodules. 

Interestingly, two NAD-dependent malic enzymes belonging to EC 1.1.1.39/40 (PHAVU_008G068700g and PHAVU_005G166400g) showed a significant increase in expression. *Arabidopsis thaliana* multiple mutants impaired in NAD-dependent malic enzyme activity were reported to alter the levels of several sugars, amino acids, to have low citrate and fumarate contents and to accumulate 2-oxoglutarate and succinate [47]. This supports a scenario in which *P. vulgaris* may sanction Fix^−^ bacteroids by succinate depletion. Of note, PHAVU_010G073700g, the common bean ortholog of the *A. thaliana* high-affinity ammonium transporter AMT2 (under nitrogen regulation in the root [48]) was found 6-fold up-regulated in *nifA* nodules. As several endopeptidases (Supplementary Table S4) had increased transcript abundance in *nifA* nodules compared to wild-type (Supplementary Figure S2a and Table S5), a simple scenario in which autophagy and proteolysis contribute to the salvage of nitrogenous compounds is plausible. 

We report here that the nodules infected by a *nifA* mutant accumulate BR relative to nodules occupied by wild-type *P. phymatum*. To our surprise, in contrast to the clear metabolomic profiles, the *nifA* nodules we sampled did not demonstrate an obvious upregulation of genes associated with BR biosynthesis. Instead, a large number (23) of the 107 *P. vulgaris* genes we could associate to steroids synthesis appeared repressed in *nifA* nodules, while only four displayed significant upregulation. It is thus possible that the increased BR content in *nifA* nodules originates from repressed regulation processes. For example, the putative brassinolide and castasterone glucosyl transferases UGT73C5 PHAVU_001G182400g, PHAVU_001G182300g and PHAVU_007G152800g showed downregulation, possibly preventing conjugation-driven BR deactivation and recycling. In fine, BR have been recognized to suppress early nodulation events such as infection thread formation and stimulate nodule initiation, and do interact with auxin signaling pathways [35]. 

We found two transcripts belonging to the auxin-responsive *GRETCHEN HAGEN* 3 (GH3) family (PHAVU_011G153400g and PHAVU_002G014400g) with 3 to 4-fold expression increase in *nifA* compared to wild-type nodules. These IAA-amido synthases potentially conjugate aspartate and other amino acids to IAA, suggesting that bacterial-derived auxins may be converted to storage forms of the hormone to regulate downstream responses. Similarly, the up-regulated PHAVU_002G038600g gene encodes a chalcone synthase homolog which, besides its pivotal role in flavonoids biosynthesis, may participate into the regulation of auxin transport. The high abundance of IAM in *nifA* nodules did not classically impact the expression of prominent auxin marker genes in our samples. Nevertheless, an ortholog of the *A. thaliana* major auxin facilitator PIN1, PHAVU_001G121100g, as well as the PIN-like auxin transporter PHAVU_010G051500g showed a decrease in relative abundance in *nifA* compared to wild-type nodules. Likewise, an ortholog of the *A. thaliana* auxin biosynthetic gene YUC6 (PHAVU_009G105400g) was also down-regulated in the mutant nodules. A handful of less-described auxin-responsive transcripts also displayed down-regulation in our *nifA* dataset, but a scenario in which the accumulation of IAM triggers a profound auxin-based reprogramming of the sampled nodule is not favored. Nonetheless, it is worth to mention that two *Arabidopsis BLADES-ON-PETIOLE* (BOP) orthologs, PHAVU_001G124100g (AtBOP1) and PHAVU_007G248000g (AtBOP2), orthologous to the *Medicago truncatula* NOOT and *Pisum sativum* COCH root identity repressors [49], were found down-regulated in *nifA* nodules. Even though the determinate *P. vulgaris* nodules cease early meristematic growth, the organogenetic and tissue identity functions of these two factors resonate with the auxin and BR homeostatic changes observed in *nifA* nodules and their aberrant clustering, which will require further attention [50]. 

In line with these results, a large subset of the putative proteins responsible for the maintenance of the autoregulation of nodulation (AON) were found to be significantly repressed in *nifA* nodules. This fabacean-specific systemic negative feedback loop normally restricts excessive nodule numbers in the plant-rhizobia interaction, presumably to balance the energy cost of hosting nitrogen-fixing bacteroids. First, the CLAVATA3/ESR (CLE)-related protein PHAVU_003G177600g (CLE12 or 13), a pre-propeptide orthologous to the *Glycine max* NIC1 and RIC1, together with the RIC2 ortholog PHAVU_011G135900g, showed significantly lower transcripts abundance in nodules occupied by the *nifA* mutant. Nitrate-induced CLE peptides were shown to suppress soybean nodule progression via the nodulation autoregulation receptor kinase GmNARK. GmNARK is homologous to *Lotus japonicus* HYPERNODULATION ABERRANT ROOT FORMATION, and both proteins are homologs of the well-described *A. thaliana* CLAVATA1 (CLV1) [51]. The *P. vulgaris* CLV1 ortholog PHAVU_011G042100g was also found significantly repressed in absence of NifA. Interestingly, another CLAVATA1-related receptor kinase-like protein (PHAVU_003G231400g/AtBAM1), required for shoot apical meristem maintenance, showed reduced expression in mutant nodules, suggesting that this putative member of the CLE-CLAVATA1 signaling pathway might play as well as a role in AON. Another gene potentially involved in AON, the *M. truncatula* ERF/AP2 transcription factor ortholog PHAVU_010G159500g, also displayed significant lower transcripts abundance in nodules colonized by the *nifA* mutant. It is plausible that the nitrogen-deprived status of *nifA* nodules represses host control over nodulation events. The *nifA* hypernodulating phenotype may thus arise from the deregulation of early nodule proliferation. 

The most prominent functional groups of down-regulated transcripts relate to carbohydrate metabolism and plant cell wall organization and dynamics (Supplementary Figure S2b and Table S6). First, key genes associated to cellulose biosynthesis and its regulation were found repressed, including homologs of cellulose synthases or well-characterized CesA-interacting proteins (Supplementary Figures S4 and S6). Second, the plethora of plant cell wall-modifying enzymes with lower expression in *nifA* nodules clearly indicate a decrease in cell elongation and overall growth in this organ. The lower growth is supported by the down-regulation of several cytoskeleton-associated transcripts known to support cell wall biogenesis (core tubulin components and tubulin- or actin-binding proteins) (Supplementary Figure S4 and Table S6). Similarly, two GO terms clusters, “Regulation of developmental growth” and “Root development” were found enriched, in line with the small size of *nifA*-colonized nodules (Supplementary Figure S2b) [18]. Several genes belonging to the GO term “Disaccharide metabolic process”, such as two trehalose 6-phosphate phosphatases, three sucrose synthases or active in sucrose catabolism (cytosolic invertase) or transport (sucrose/H+ symporter) together with a sugar isomerase, a hexose/H+ symporter and three nucleotide-sugar transporters showed decreased expression in *nifA* nodules (Supplementary Figure S4 and Table S6). This strongly suggests that carbon supply to non-fixing symbionts might be actively reduced. In contrast, we found four STP-like Major facilitator superfamily proteins significantly overexpressed in *nifA* Fix^−^ nodules (Supplementary Tables S4 and S6). These sugars/H+ symporters control monosaccharides homeostasis and the over-expression of STP13 in *A. thaliana* improved growth and nitrogen use [52]. It is tempting to speculate that hexoses recovered from the cell wall turnover could be diverted from the inefficient *nifA* nodules. 

The nodules colonized by the *nifA* mutant displayed a strong reduction in flavonoids contents compared to wild-type-colonized ones. This reduction is also reflected in the overall down-regulation of genes involved in the biosynthesis and conversion of this class of compounds. In particular, the strongly reduced transcripts abundance of three putative CYP81 family hydroxylases (Supplementary Table S4) seems to correlate with the decrease in downward isoflavonoids (Supplementary Figure S3a and Table S6). Similarly, the CYP98 family monooxygenase PHAVU_001G117900g, the caffeoyl-CoA O-methyltransferase PHAVU_003G242400g and the shikimate O-hydroxycinnamoyltransferase PHAVU_005G183200g show a coordinated reduction in expression, presumably hindering *p*-coumaroyl-CoA conversion. The same expression kinetics were found for a dozen of genes involved in phenylpropanoids biosynthesis.

Nitrogen is essential to amino and nucleic acids synthesis in plants. As expected, many repressed genes clustered in functional GO terms related to organonitrogen usage, amine, purine and amino acids metabolism under *nifA*-triggered nitrogen limitation. We could map a total of 61 *P. vulgaris* genes and metabolites to the KEGG pathway “Biosynthesis of amino acids” and observed a strong congruence between down-regulated metabolic genes in *nifA* nodules (Supplementary Figure S2b). Interestingly, *P. vulgaris* seems to participate in the net aspartate accumulation observed in nodules occupied by the *nifA* mutant as the asparagine synthase PHAVU_006G069300g, the asparaginase PHAVU_001G025000g or the aspartate transporter PHAVU_001G077000g showed marked reductions in expression (Supplementary Figure S3b). This further highlights the defective nitrogen assimilation by the plant host in *nifA* nodules. On the other hand, the glutamate synthase PHAVU_001G076400g and glutamate dehydrogenase PHAVU_004G080200g displayed lower transcription in nodules occupied by the mutant, thus the lower abundance of glutamate, glutamine and 2-oxoglutarate may directly result in the observed depletion in succinate in these tissues (Supplementary Figure S3b). The reduced expression of a functional module comprising the urate hydroxylase PHAVU_007G234300g, the hydroxyisourate hydrolase PHAVU_010G034000g, the 2-oxo-4-hydroxy-4-carboxy-5-ureidoimidazoline decarboxylase PHAVU_010G034000g and the allantoinase PHAVU_006G186700g could account for the observed decrease in allantoin levels. 

Taken together, our data suggest that the bean nitrogen assimilation program is greatly affected in *nifA*-colonized nodules, as exemplified by the drastic down-regulation of the nitrate reductase PHAVU_009G121000g or of the nitrate-inducible, GARP-type transcriptional repressor PHAVU_003G028000g. Similarly, the *A. thaliana* bZIP domain transcription factor NITRATE REGULATORY GENE (NRG) ortholog PHAVU_010G050100g was repressed in absence of NifA.

### 2.4. The nifA Mutant Strain Produces a Higher Amount of Auxin Compounds

**Figure 4:**
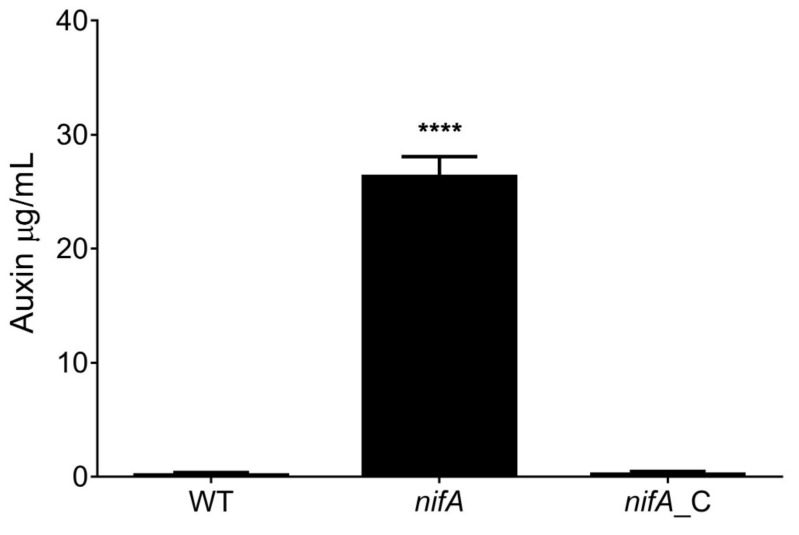
Auxinic compound production by *P. phymatum* STM815 (WT), *nifA* insertional mutant (*nifA*) and the complemented strain (*nifA_*C). Strains were grown in LB medium without salt for 16 h and the release of auxins was measured by colorimetry using indole−3-acetate (IAA) as standard. The experiment was performed with three biological replicates. Error bars indicate error of the mean (SEM). Significance was calculated by ANOVA, Tukey’s test (****: *p-*value < 0.0001).

The decrease in the amount of the auxin precursor tryptophan and the increase in indole-acetamide (IAM) as well as the increase in transcript levels of Bphy_7768 coding for a IAM hydrolase (*iaaH*), suggested that *P. phymatum* NifA is involved in the regulation of auxin levels inside the nodule. In order to investigate a possible influence of NifA in auxins production, the amount of secreted indolics was measured in wild-type, *nifA* mutant and *nifA* complemented strain grown in complex liquid medium. After growing the three strains for 16 h, the supernatant was collected and the amount of auxins determined. Our quantification showed that the *nifA* mutant exported 180-fold more auxins in free-living conditions compared to the wild-type, i.e., around 26 µg/mL. The amount of auxinic compounds produced by the mutant could be restored to wild-type levels by providing *nifA* on a plasmid (Figure 4). This result suggests that NifA negatively controls auxin production by the microsymbiont, not only that inhabiting root nodules as suggested by nodules metabolomes but also in free-living conditions. Using the colorimetric method, the identity of the overproduced auxin cannot be determined more precisely. However, based on our metabolomics and transcriptomics data, we speculate that the main auxinic product could be IAM and/or IAA itself. Indeed, the data suggest a statistically significant accumulation of IAM and indole-3-acetaldehyde as well as an increased transcription of Bphy_7768 (*iaaH*), coding for the enzyme that hydrolyzes IAM into IAA. IAA is the main auxin in plants [53] and, interestingly, the overproduction of IAA via the IAM pathway in *S. meliloti* strain 1021 positively affected nodulation in *Medicago* plants, while the IAA overproducer *Rhizobium leguminosarum bv. phaseoli*, did not influence nodule number in *P. vulgaris* [54]. In absence of alterations in the abundance of IAA-biosynthetic plant transcripts, we assume that the observed auxin accumulation originated from the peribacteroid space (PBS). Even though auxin peribacteroid membrane IAA importers have been suggested [55], no IAA exporters have been reported to date. However, the PBS in *M. truncatula*/*S. meliloti* nodules was found acidic; if low pH also occurs in bean PBS, the diffusion of protonated IAA through the peribacteroid membrane would be facilitated [56].

### 2.5. The nifA Mutant Strain Is Affected in Growth on Plates Containing Succinate, Malate, Glutamine and Glutamate as Only Carbon Sources 

**Figure 5:**
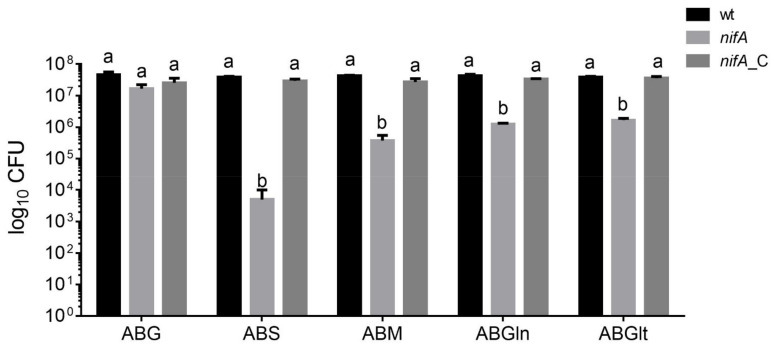
Growth of *P. phymatum* wild-type (wt), *nifA* mutant strain *(nifA*) and its complemented strain (*nifA_*C) in AB minimal media with different carbon sources. First, the strains were grown in full media for 16 h, then the cells were washed and the OD_600_ adjusted to 0.05. Next, serial dilutions were spotted on LB without salt rich media (LB) and AB media supplemented with 10 mM of glucose (ABG) as a control, AB supplemented with 15 mM of the C_4_-dicarboxylate succinate (ABS) or malate (ABM) and AB supplemented with 12 mM of the amino acids glutamine (ABGln) or glutamate (ABGlt). The growth of the strains is shown as Log_10_ of the colony-forming units (CFU). At least three biological replicates were performed in this analysis. Error bars indicate the standard error of the mean (SEM). For each condition, values with the same letter (a, b) are not significantly different, while those with different letters are (ANOVA and Tukey’s test using a *p-*value < 0.05).

Our metabolomics data showed a decrease in the C_4_-dicarboxylate succinate and in the amino acid glutamine and glutamate in nodules occupied by the *nifA* mutant compared to wild-type nodules. Moreover, the RNA-Seq analysis showed that *P. phymatum* C_4_-dicarboxylate transporter DctA (Bphy_0225) encoding gene was down-regulated in *nifA* nodules (Figure 3f), although did not pass our threshold settings (*p*-value = 0.024). We therefore investigated the viability of wild-type, *nifA* mutant and complemented strain on several carbon sources. For this, the same number of cells (10^7^ cells) previously grown in liquid complex media, were plated in minimal media with different carbon sources. While the *nifA* mutant was not affected in growth in complex medium (LB without salt) and in minimal medium containing the C_6_ compounds glucose (ABG), the *nifA* mutant displayed up to 100-fold reduction in cell number when grown in minimal medium with the C_4_-dicarboxylates succinate and malate and this defect was complemented by providing *nifA in trans* (Figure 5). It is known that C_4_-dicarboxylates play a key role during symbiosis in rhizobia and that blocking the dicarboxylate transport (Dct) system leads to a nitrogen fixation failure [3]. These results suggest that NifA affect the transport of succinate and malate by regulating the Dct system in free-living conditions and during symbiosis. When we grew the cells in presence of glutamine and glutamate as only carbon sources, the growth of the *nifA* mutant was also found reduced one log, suggesting the involvement of NifA during the assimilation of these amino acids (Figure 5). The protonated form of ammonia, which is formed from the reduction of atmospheric nitrogen, is known to bind to glutamate in order to produce glutamine that is distributed and assimilated by plant cells [4].

### 2.6. NifA Plays a Role in Osmotic Tolerance 

**Figure 6:**
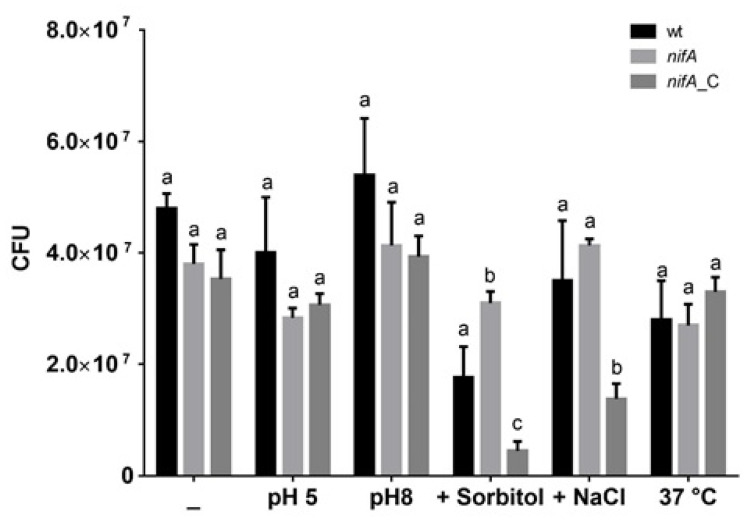
Sensitivity of *P. phymatum* wild-type (wt), *nifA* mutant strain (*nifA*) and its complemented strain (*nifA_*C) in LB without salt complex media subjected to different stress. Grown cultures of the three strains were adjusted to an OD_600_ of 0.05, serial dilutions were spotted on the LB without salt plate (−), with pH adjusted to 5 (pH 5) or to 8 (pH 8), supplemented with 400 mM of D-sorbitol (+Sorbitol) or with 200 mM of NaCl (+NaCl) or on LB without salt plates incubated at 37 °C. The survival of the strains is shown as the colony-forming units (CFU). At least three biological replicates were performed in this analysis. Error bars indicate the standard error of the mean (SEM). For each condition, values with the same letter (a, b, c) are not significantly different, while those with different letters are (ANOVA and Tukey’s test using a *p-*value < 0.05).

Due to the observed up-regulation of several genes involved in the response to stress in *nifA* mutant bacteroids (the sigma factor RpoH, a catalase, an alkyl peroxidase), we compared the sensitivity of *P. phymatum* wild-type, *nifA* mutant and complemented strains to different kinds of stress such as acidic and alkaline pH (pH 5 and pH 8, respectively), osmolarity (400 mM of D-sorbitol), salinity (200 mM of NaCl), oxidative (35%, 7% and 3.5% H_2_O_2_) and temperature (incubation at 37 °C). The survival of these strains was tested by spotting several dilutions on the respective plates modified according to the stress condition to be tested. After 40 h incubation, the three *P. phymatum* strains did not show any significant difference in growth under low or high pH, salinity or temperature stress. However, the *P. phymatum nifA* mutant was more resistant to osmotic stress compared to the wild-type and the complemented strain (Figure 6). This phenotype could be attributed to the up-regulation of the expression of *rpoH*, coding for the sigma factor known to play a key role in the adaptation to heat-shock, osmotic and oxidative stress [44]. Nevertheless, all *P. phymatum* strains were found to be similarly sensitive to H_2_O_2_ (data not shown).

## 3. Materials and Methods

### 3.1. Bacterial Strains, Media and Cultivation

The primers, plasmids and bacterial strains used in this work are listed in the Supplementary Materials (Supplementary Table S7). *Escherichia coli* strains were habitually grown under aerobic conditions at 37 °C in Luria-Bertani liquid medium (LB) [57], while *P. phymatum* STM815 strains were aerobically cultured at 28 °C in a modified LB liquid medium without salt. Where necessary, the antibiotics kanamycin (25 µg/mL for *E. coli* and 50 µg/mL for *P. phymatum*) and/or chloramphenicol (80 µg/mL for *P. phymatum*) were used. 

### 3.2. Plant Growth Conditions

The surface-sterilization of the common bean seed (*Phaseolus vulgaris,* cv. Negro Jamapa) was carried out by soaking in absolute ethanol for five minutes followed by another five minutes of soaking in 35% H_2_O_2_. Seeds were washed several times with sterile deionized water and were placed on 0.8% agarose plates [58]. After 48 h of germination in the dark at 28 °C, the sprouted seeds were transferred into autoclaved amber yogurt jars containing nitrogen-free Jensen medium and vermiculite (VTT-Group, Muttenz, Switzerland) [59]. The cultured bacterial cells were washed and inoculated on the germinated seeds as previously described [18]. The inoculated plants were grown for 21 days in a green-house under the conditions: 22/25 °C night/day temperatures with 16 h light (160 µmol m^−2^ s^−1^) and a constant humidity of 60%. Inoculated and non-inoculated plants were watered with sterile deionized water.

### 3.3. Metabolite Extraction and Data Analysis

Metabolite abundances were compared between *P. vulgaris* root nodules infected with *P. phymatum* strains (wild-type or *nifA* insertional mutant). The metabolites extracted from the mature part of the uninfected *P. vulgaris* roots were used as root baseline to produce Figure 2. Samples of three biological replicates (plants inoculated with three *P. phymatum* biological replicates) two technical replicates each, per strain (*P. phymatum* wild-type and *nifA* mutant) were analyzed. After 21 days, all root nodules present on the root system were collected and flash-frozen in liquid nitrogen. The correct identity of the strains present in the nodules was verified by plating on selective media and PCR. One plant at the time was extracted from the soil and immediately processed. For inoculated plant each nodule was taken and immediately flash frozen. Approximately 30 mg of nodules from two plants per sample were used for the extraction of metabolites using cold methanol method as previously described [25]. The uninfected roots (30 mg) were processed in the same way as the nodules and as has been carried out in previous studies [19,25,26]. Next, the hydrophilic methanol extracts were injected in an Agilent 6550 QTOF instrument (Agilent Technologies, Santa Clara, CA, USA) and analyzed by non-targeted flow injection-time-of-flight mass spectrometry, as shown before [60]. A total of 437 ions with distinct m/z were matched to expected deprotonated molecules which were then statistically analyzed with a two-tailed, heteroscedastic *t*-test using a false discovery rate (FDR) correction according to Storey and Tibshirani [61]. Metabolites showing statistically significant increased/decreased levels were selected using the following criteria: fold change (FC) threshold of log_2_ ≥0.5 or ≤−0.5 and *q*-value ≤ 0.01. All the metabolomics raw data, including ions, annotations and intensities, are listed in the Supplementary Table S8. The integrated metabolic pathway analysis was performed in MetaboAnalyst 5.0 [62] using KEGG pathways as a reference [63].

### 3.4. RNA-Sequencing and Data Processing

Samples of root nodule induced by *P. phymatum* wild-type or mutant strains were flash-frozen in liquid nitrogen and the extraction of plant and bacterial RNA was carried out using a modified hot acid phenol protocol [64]. Two independent biological replicates were analyzed per condition. The DNase treatment for genomic DNA (gDNA) removal and the RNA quality check were performed as reported earlier [65]. Plant ribosomal RNA (rRNA) elimination was carried out using the Ribo-zero™ Plant-Seed/Root kit (Epicentre, Madison, DC, USA) [18]. After RNA quantification, the cDNA libraries were synthesized and purified with the Encore Complete Prokaryotic RNA-Seq DR Multiplex System (NuGEN, San Carlos, CA, USA), and quantified by capillary electrophoresis using a TapeStation (Agilent Technologies) [18]. The libraries were sequenced (single end and 125 base-pair) with a HiSeq2500 instrument (Illumina, San Diego, CA, USA) [18]. The obtained reads were trimmed to 70 bp, processed and mapped to the *P. phymatum* STM815 genome (NC_010622.1, NC_010623.1, NC_010625.1 and NC_010627.1) [11] or to the *P. vulgaris* genome (NC_023759.1, NC_023758.1, NC_023757.1, NC_023756.1, NC_023755.1, NC_023754.1, NC_023753.1, NC_023752.1, NC_023751.1, NC_023750.1, NC_023749.1) [66]. To achieve this, CLC Genomics Workbench v11.0 (QIAGEN CLC bio, Aarhus, Denmark) was employed, allowing up to two mismatches per read. The statistical analysis for the differential expression of the reads uniquely mapped to the genome was conducted with the DESeq R-package version 1.38.0 [67]. For the DESeq analysis, the top 479 and 1572 significantly regulated genes from *P. phymatum* and *P. vulgaris*, respectively (ranked by ascending *p-*value), were selected. A FC threshold of log_2_ ≥1 or ≤−1 was applied with a *p*-value ≤ 0.02. Functional enrichment in gene ontology terms was carried out using the Cytoscape 3.5 plugin ClueGo v2.5.8 with default settings. The complete list of all *P. phymatum* and *P. vulgaris* genes and their log_2_ FC by comparing their expression in the *nifA* mutant induced nodules versus their expression in the wild-type nodules can be found in the Supplementary Tables S2 and S3.

### 3.5. Construction of P. phymatum STM815 Complemented Strains

*P. phymatum* STM815 genomic DNA was isolated using the GenElute™ Bacterial Genome DNA Kit (Sigma-Aldrich, St. Louis, MO, USA), while plasmid DNA from *E. coli* strains was obtained by using the QIAprep Spin Miniprep Kit (Qiagen, Hilden, Germany). To generate the complemented strain of the previously constructed *nifA* insertional mutant [18], the *P. phymatum nifA* (Bphy_7728) was amplified by PCR using the primers nifA_comp_F_HindIII and nifA_comp_R_XbaI. The 2043 bp-long product was digested with the restriction enzymes *Hind*III and *Xba*I and ligated into the pBBR1MCS-2. The constructed plasmid was then mobilized to the *nifA* mutant strain by triparental mating. The sequence of the constructed plasmid was confirmed by sequencing at Microsynth (Balgach, St. Gallen, Switzerland). 

### 3.6. Auxin Production

The production of auxinic compounds by *P. phymatum* STM815 wild-type and *nifA* mutant strains was quantified by a modified colorimetric assay from Gravel and colleagues [68]. Precultures were grown aerobically at 28 °C in LB without salt with the corresponding antibiotics, washed twice and inoculated into 100 mL flasks containing 20 mL of LB without salt with an initial OD_600_ of 0.05. Cultures were incubated at 28 °C with 180 rpm shacking for 16 h until all the strains reached an OD_600_ of 4. Two mL of culture was then centrifuged (5000 rpm, 5 min) and one mL of the supernatant was mixed with two mL of Salkowski’s reagent [69]. The mixture was incubated at room temperature in the dark for 20 min and the absorbance was measured at 535 nm. To quantify extracellular auxinic compounds, a 50 µg/mL of indole-3-acetic acid (IAA) (Sigma-Aldrich, Buchs, St. Gallen, Switzerland) solution was diluted in LB without salt to draw the standard curve. This experiment was conducted in three biological replicates. 

### 3.7. Carbon Source and Stress Survival Assay

The carbon source assimilation and the stress tests of the different *P. phymatum* strains were assayed on agar plates containing different media, as described below. Cell growth with different carbon sources was tested on the AB minimal media [70] agar plates supplemented with: 15 mM of succinate or malate; 10 mM of glucose; 12 mM of glutamine or glutamate. Cell sensitivity to acidic or alkaline pH, osmotic or salt stress were tested on LB without salt medium plates with following modifications: pH adjusted to either 5 or 8, supplemented with 400 mM of D-sorbitol or supplemented with 200 mM of NaCl, respectively. The bacterial precultures were grown as mentioned above (see Section 3.6). Cells were collected by centrifugation, washed twice with 10 mM MgSO_4_ and adjusted to OD_600_ 0.05. Then, 10-fold serial dilutions were performed. Five µL of each dilution of the strains were spotted on above mentioned plates and a standard LB without salt plate as a control. The plates were incubated at 28 °C until the colonies were countable. The cell viability in each test was determined by the colony-forming units (CFU). Similarly, the temperature stress was assayed on standard LB without salt plates and incubated at 37 °C.

### 3.8. Statistical Analysis

The statistical analysis for all the phenotypical tests (auxin production, carbon-source utilization and stress susceptibility assay) were performed with GraphPad Prism 6.0. The significant difference in auxin production was assessed using a one-way ANOVA and Tukey’s test with a *p*-value < 0.05. To analyze the cell viability using different carbon sources and under different stress factors, the CFU of three strains were analyzed with ANOVA and Tukey’s test with *p-*value < 0.05 for each condition. 

## 4. Conclusions

In this study, we integrated metabolome and dual RNA-seq transcriptome data and thereby report for the first time the changes occurring in both symbiotic partners in the absence of NifA. Apart from the classical nitrogen deprivation signs observed in non-fixing bacteroids, we show that in the absence of *P. phymatum* NifA the level of several metabolites involved in BR and auxin biosynthesis was highly increased. Moreover, several *P. phymatum* genes related to the stress response showed an increased expression or amount in *nifA* mutant bacteroids. In contrast to the increased amount of auxins observed in *nifA* nodules, the plant host did not demonstrate canonical systemic or local auxin responses in these tissues. We speculate that the absence of NifA during the nodule initiation or early development mediates the emergence of clustered nodules by unbalancing BR-auxin homeostasis. Moreover, the hypernodulation phenotypes of *P. vulgaris* plants inoculated with the *nifA* mutant could be explained by the down-regulation of transcripts involved in AON. Further, in the absence of NifA, despite a marked nitrogen-limitation response, *P. vulgaris* nodules did not mount an efficient immune response to the mutant bacteria. These results differ from the ones we previously obtained with soybean nodules infected by a *B. diazoefficiens* NifA mutant, where a strong defense reaction was detected in form of the production of high level of phytoalexins. Finally, this study revealed that *P. phymatum* NifA induces not only the changes that are in part common to alpha-rhizobial symbiosis, but also other changes that are specific for *P. phymatum*–*P. vulgaris* symbiosis. Whether these NifA-dependent changes are conserved in other beta-rhizobial symbionts still remains to be elucidated, as well as the molecular mechanisms behind this regulatory network.

## Figures and Tables

**Table 1:** List of the 142 genes located in *P. phymatum* STM815 symbiotic plasmid (pBPHY02) that showed a differential expression in common bean nodules infected with *nifA* mutant strain compared to the wild-type nodules (abs[log_2_(fold-change)] ≥ 0.5 and *p*-value ≤ 0.02).

Locus ID ^1^	Description ^1^	Old Locus ID ^1^	Gene Name ^1^	log_2_FC (*nifA* vs. wt) ^2^
**Genes up-regulated in *nifA* mutant bacteroids**				
BPHY_RS41160	hypothetical protein	Bphy_7816		4.47
BPHY_RS38115	hypothetical protein	Bphy_7726		2.53
BPHY_RS38105	ATP-grasp domain-containing protein	Bphy_7724		2.26
BPHY_RS36585	DNA-binding protein	Bphy_7384		2.24
BPHY_RS38320	indoleacetamide hydrolase	Bphy_7768	*iaaH*	1.69
**Genes down-regulated in *nifA* mutant bacteroids**				
BPHY_RS37800	branched-chain amino acid ABC transporter substrate-binding protein	Bphy_7652		−2.10
BPHY_RS37125	GntR family transcriptional regulator	Bphy_7505		−2.18
BPHY_RS37205	hypothetical protein	Bphy_7521		−2.25
BPHY_RS37050	biosynthetic-type acetolactate synthase large subunit	Bphy_7490	*ilvB*	−2.27
BPHY_RS37120	ABC transporter substrate-binding protein	Bphy_7504		−2.32
BPHY_RS38545	iron-sulfur cluster assembly accessory protein	Bphy_7815		−2.33
BPHY_RS38560	porin	Bphy_7819		−2.47
BPHY_RS37795	aminotransferase class III-fold pyridoxal phosphate-dependent enzyme	Bphy_7651		−2.51
BPHY_RS37925	hypothetical protein	Bphy_7682		−2.52
BPHY_RS37805	IS630 family transposase	Bphy_7653		−2.57
BPHY_RS37025	DMT family transporter	Bphy_7485		−2.58
BPHY_RS38285	carbon starvation protein A	Bphy_7759		−2.62
BPHY_RS37790	M81 family peptidase	Bphy_7649		−2.71
BPHY_RS37915	hypothetical protein	Bphy_7680		−2.84
BPHY_RS41075	hypothetical protein	Bphy_7694		−2.95
BPHY_RS37775	ABC transporter permease	Bphy_7646		−3.00
BPHY_RS36465	hypothetical protein	Bphy_7358		−3.01
BPHY_RS37930	hypothetical protein			−3.13
BPHY_RS38520	nitrogenase molybdenum-iron protein alpha chain	Bphy_7809		−3.16
BPHY_RS37765	ABC transporter substrate-binding protein	Bphy_7644		−3.16
BPHY_RS38135	nitrogenase iron-molybdenum cofactor biosynthesis protein	Bphy_7730	*nifN*	−3.16
BPHY_RS37090	pyridoxal-phosphate dependent enzyme	Bphy_7498		−3.19
BPHY_RS41155	hypothetical protein	Bphy_7814		−3.27
BPHY_RS41070	cytolethal distending toxin subunit B family protein	Bphy_7691		−3.30
BPHY_RS37780	ABC transporter ATP-binding protein	Bphy_7647		−3.40
BPHY_RS40855	IS110 family transposase	Bphy_7484		−3.40
BPHY_RS37770	ABC transporter permease	Bphy_7645		−3.49
BPHY_RS37045	ISL3 family transposase	Bphy_7489		−3.60
BPHY_RS37920	hypothetical protein	Bphy_7681		−3.62
BPHY_RS38485	electron transfer flavoprotein subunit alpha/FixB family protein	Bphy_7803	*fixB*	−3.63
BPHY_RS41535	IS3 family transposase			−3.63
BPHY_RS36015	HyaD/HybD family hydrogenase maturation endopeptidase	Bphy_7262	*hybD*	−3.70
BPHY_RS36485	hypothetical protein			−3.83
BPHY_RS41135	electron transfer flavoprotein alpha/beta-subunit	Bphy_7804		−3.90
BPHY_RS36645	alpha/beta fold hydrolase	Bphy_7397		−3.91
BPHY_RS38310	cytochrome P450	Bphy_7766		−4.00
BPHY_RS40820	IS5/IS1182 family transposase			−4.06
BPHY_RS38020	radical SAM protein	Bphy_7706		−4.07
BPHY_RS37785	ABC transporter ATP-binding protein	Bphy_7648		−4.10
BPHY_RS37970	7-carboxy-7-deazaguanine synthase QueE	Bphy_7690		−4.11
BPHY_RS37005	GntR family transcriptional regulator	Bphy_7481		−4.44
BPHY_RS36735	ArgP/LysG family DNA-binding transcriptional regulator	Bphy_7420		−4.51
BPHY_RS37085	Lrp/AsnC family transcriptional regulator	Bphy_7497		−4.58
BPHY_RS38495	FAD-binding oxidoreductase	Bphy_7805		−4.62
BPHY_RS36730	sodium:solute symporter	Bphy_7419		−4.66
BPHY_RS35975	carbamoyltransferase	Bphy_7254	*hypF*	−4.74
BPHY_RS38270	hypothetical protein			−4.78
BPHY_RS37910	IS6 family transposase	Bphy_7679		−4.80
BPHY_RS36725	fatty acid desaturase	Bphy_7418		−4.87
BPHY_RS38515	nitrogenase iron protein	Bphy_7808	*nifH*	−4.88
BPHY_RS36010	HypC/HybG/HupF family hydrogenase formation chaperone	Bphy_7261	*hypC*	−4.95
BPHY_RS38025	glycosyltransferase	Bphy_7707		−4.98
BPHY_RS38365	putative nitrogen fixation protein	Bphy_7777	*nifT*	−5.01
BPHY_RS35985	hydrogenase maturation nickel metallochaperone	Bphy_7256	*hypA*	−5.14
BPHY_RS37115	histidine ABC transporter permease	Bphy_7503	*hisQ*	−5.14
BPHY_RS38425	class I SAM-dependent methyltransferase	Bphy_7790		−5.14
BPHY_RS36060	hydrogenase expression/formation protein	Bphy_7271	*hypE*	−5.17
BPHY_RS37065	porin	Bphy_7493		−5.33
BPHY_RS38140	nitrogen fixation protein	Bphy_7731	*nifX*	−5.35
BPHY_RS36005	hydrogenase	Bphy_7260	*hyaE*	−5.43
BPHY_RS36050	hypothetical protein			−5.51
BPHY_RS36020	Ni/Fe-hydrogenase, b-type cytochrome subunit	Bphy_7263	*cybH*	−5.63
BPHY_RS36740	SDR family oxidoreductase	Bphy_7421		−5.68
BPHY_RS40825	glutamine amidotransferase class-II	Bphy_7471		−5.75
BPHY_RS38160	hypothetical protein	Bphy_7735		−5.82
BPHY_RS37075	hypothetical protein	Bphy_7495		−5.83
BPHY_RS37110	histidine ABC transporter permease	Bphy_7502	*hisM*	−5.83
BPHY_RS41065	cupin	Bphy_7689		−5.83
BPHY_RS38225	hypothetical protein	Bphy_7748		−5.88
BPHY_RS38130	nitrogenase iron-molybdenum cofactor biosynthesis protein	Bphy_7729	*nifE*	−5.89
BPHY_RS37735	NAD-dependent epimerase/dehydratase family protein	Bphy_7636		−5.92
BPHY_RS41080	AraC family transcriptional regulator	Bphy_7704		−5.92
BPHY_RS37080	MmgE/PrpD family protein	Bphy_7496		−5.97
BPHY_RS37105	ATP-binding cassette domain-containing protein	Bphy_7501		−6.13
BPHY_RS36745	histidinol dehydrogenase	Bphy_7422	*hisD*	−6.13
BPHY_RS36000	hydrogenase expression/formation protein	Bphy_7259		−6.16
BPHY_RS35990	HupK protein	Bphy_7257	*hupK*	−6.18
BPHY_RS41530	transposase	Bphy_7402		−6.22
BPHY_RS37965	class I SAM-dependent methyltransferase	Bphy_7688		−6.25
BPHY_RS36055	cysteine hydrolase	Bphy_7270		−6.29
BPHY_RS37070	ABC transporter substrate-binding protein	Bphy_7494		−6.33
BPHY_RS40990	IS5/IS1182 family transposase	Bphy_7635		−6.34
BPHY_RS37100	aminopeptidase P family protein	Bphy_7500		−6.59
BPHY_RS38155	nitrogen fixation protein	Bphy_7734	*nifQ*	−6.59
BPHY_RS35995	[NiFe]-hydrogenase assembly, chaperone	Bphy_7258	*hybE*	−6.61
BPHY_RS41545	FAD-dependent oxidoreductase			−6.68
BPHY_RS41140	hypothetical protein	Bphy_7806		−6.82
BPHY_RS41095	hypothetical protein	Bphy_7746		−6.85
BPHY_RS39040	pyridoxal-phosphate dependent enzyme	Bphy_7479		−6.85
BPHY_RS38150	ferredoxin III, nif-specific	Bphy_7733	*fdxB*	−6.86
BPHY_RS36650	IS5/IS1182 family transposase	Bphy_7398		−6.90
BPHY_RS36025	nickel-dependent hydrogenase large subunit	Bphy_7264		−6.96
BPHY_RS38145	hypothetical protein	Bphy_7732		−7.01
BPHY_RS38245	ankyrin repeat domain-containing protein	Bphy_7751		−7.11
BPHY_RS38170	protein FixC	Bphy_7737	*fixC*	−7.11
BPHY_RS37030	ISL3 family transposase	Bphy_7486		−7.17
BPHY_RS37740	SDR family NAD(P)-dependent oxidoreductase	Bphy_7637		−7.21
BPHY_RS38230	IS630 family transposase	Bphy_7749		−7.34
BPHY_RS38255	nitrogenase iron protein	Bphy_7753	*nifH*	−7.40
BPHY_RS36670	aldehyde dehydrogenase family protein	Bphy_7406		−7.41
BPHY_RS38525	LysR family transcriptional regulator	Bphy_7810		−7.42
BPHY_RS36065	carbamoyltransferase	Bphy_7272	*hypF*	−7.47
BPHY_RS36035	hypothetical protein			−7.51
BPHY_RS36675	maltose alpha-D-glucosyltransferase	Bphy_7407	*treS*	−7.53
BPHY_RS38220	putative nitrogen fixation protein	Bphy_7747	*nifT*	−7.55
BPHY_RS36040	diaminobutyrate-2-oxoglutarate transaminase	Bphy_7266	*ectB*	−7.58
BPHY_RS38265	nitrogenase molybdenum-iron protein subunit beta	Bphy_7755	*nifK*	−7.67
BPHY_RS38240	zinc ribbon domain-containing protein	Bphy_7750		−7.72
BPHY_RS38185	nitrogenase stabilizing/protective protein	Bphy_7740	*nifW*	−7.78
BPHY_RS36655	group II intron reverse transcriptase/maturase	Bphy_7399	*ltrA*	−7.78
BPHY_RS36045	GNAT family N-acetyltransferase	Bphy_7268		−8.06
BPHY_RS40995	IS5/IS1182 family transposase	Bphy_7638		−8.07
BPHY_RS37730	multi anti extrusion protein	Bphy_7634	*matE*	−8.21
BPHY_RS37000	ISL3 family transposase	Bphy_7480		−8.22
BPHY_RS36990	GHMP kinase	Bphy_7478		−8.24
BPHY_RS38190	homocitrate synthase	Bphy_7741	*nifV*	−8.28
BPHY_RS37725	D-alanine-D-alanine ligase	Bphy_7633		−8.45
BPHY_RS38165	ferredoxin	Bphy_7736	*fixX*	−8.93
BPHY_RS38175	electron transfer flavoprotein subunit alpha/FixB family protein	Bphy_7738	*fixB*	−9.16
BPHY_RS36680	ABC transporter ATP-binding protein	Bphy_7408		−9.33
BPHY_RS38195	nitrogenase cofactor biosynthesis protein	Bphy_7742	*nifB*	−9.83
BPHY_RS38260	nitrogenase molybdenum-iron protein alpha chain	Bphy_7754	*nifD*	−10.05
BPHY_RS36030	twin-arginine translocation signal domain-containing protein	Bphy_7265	*hydA*	−10.25
BPHY_RS38180	electron transfer flavoprotein beta subunit/FixA family protein	Bphy_7739	*fixA*	−10.52
BPHY_RS38505	hypothetical protein	Bphy_7807		−11.11
BPHY_RS38250	hypothetical protein	Bphy_7752		−11.54
BPHY_RS40755	IS66 family transposase	Bphy_7404		NA
BPHY_RS37010	IS3 family transposase	Bphy_7482		NA
BPHY_RS36905	glycolate oxidase subunit G	Bphy_7454	*glcD*	NA
BPHY_RS41325	diaminobutyrate-2-oxoglutarate transaminase	Bphy_7273		NA
BPHY_RS38360	nitrogen fixation protein	Bphy_7776	*nifZ*	NA
BPHY_RS37060	hypothetical protein			NA
BPHY_RS38235	hypothetical protein			NA
BPHY_RS35970	HypC/HybG/HupF family hydrogenase formation chaperone	Bphy_7253	*hypC*	NA
BPHY_RS38210	nitrogen fixation protein	Bphy_7745	*nifZ*	NA
BPHY_RS40615	hypothetical protein	Bphy_7267		NA
BPHY_RS40750	hypothetical protein			NA

^1^ Locus tag, gene description, old locus tag and gene name is given according to the RefSeq files (NC_010622.1, NC_010623.1, NC_010625.1, NC_010627.1); ^2^ Log_2_ Fold Change (FC) of transcriptional level, comparing *nifA* mutant with wild-type strain nodules; NA, the read number of the *nifA* nodules was 0. ATP, Adenosine Tri-Phosphate; ABC, ATP-Binding Cassette; IS, Insertion Sequence; DMT, Drug/Metabolite Transporter; SAM, S-Adenosyl Methionine; SDR, Short-Chain Dehydrogenase Reductase; FAD, Flavin Adenine Dinucleotide; NAD(P), Nicotinamide adenine dinucleotide phosphate; GNAT, Gcn5-related N-acetyltransferases.

## Data Availability

The RNA-sequencing raw data files obtain in this work are accessible through the GEO Series accession number GSE176287.

## Supplementary Materials

The following are available online at https://www.mdpi.com/article/10.3390/metabo11070455/s1, Figure S1: Modified pathways from (a) alanine, aspartate and glutamate metabolism (https://www.genome.jp/pathway/bph00250; accessed on 26 June 2021) and (b) tryptophan metabolism (https://www.genome.jp/pathway/bph00380; accessed on 26 June 2021) obtained by KEGG Mapper, metabolites less accumulated and *P. phymatum* genes down-regulated in the *nifA* mutant nodules compared to wild-type nodules, are represented in blue while the metabolites more accumulated and genes up-regulated are represented in red, Figure S2: Functional group network of differentially expressed *P. vulgaris* genes in nodules occupied by the *P. phymatum* *nifA* mutant compared to wild-type nodules: (a) up-regulated in bean nodules colonized by *P. phymatum* *nifA* mutant compared to wild-type and (b) down-regulated in *P. phymatum* *nifA* mutant compared to wild-type, disc size represents “Biological process” GO terms group p-value significance corrected with the Bonferroni step down method, Figure S3: Modified pathways from (a) isoflavonoids biosynthesis (https://www.genome.jp/pathway/pvu00943; accessed on 26 June 2021) and (b) alanine, aspartate and glutamate metabolism (https://www.genome.jp/pathway/pvu00250; accessed on 26 June 2021) obtained by KEGG Mapper, metabolites less accumulated and *P. vulgaris* genes down-regulated in the *P. phymatum* *nifA* mutant nodules compared to wild-type nodules, are represented in blue while the metabolites more accumulated and genes up-regulated are represented in red, Table S1: List of all the metabolites detected in *P. phymatum* *nifA* nodules compared to wild-type nodules, Table S2: List of all *P. phymatum* genes with their differential expression comparing bean nodules inoculated with *nifA* mutant to wild-type nodules, Table S3: List of all *P. vulgaris* genes with their differential expression comparing nodules inoculated with *P. phymatum* *nifA* mutant to wild-type, Table S4: Selection of *P. vulgaris* genes differentially expressed in the transcriptome analysis and discussed in the main text, Table S5: GO pathway specific terms analysis for up-regulated *P. vulgaris* genes from nodules colonized by *P. phymatum* *nifA* mutant compared to wild-type, Table S6: GO pathway specific terms analysis for down-regulated *P. vulgaris* genes from nodules colonized by *P. phymatum* *nifA* mutant compared to wild-type, Table S7: Bacterial strains, plasmids and primers used in this work, Table S8: Raw metabolomics data including injections (see sheet “injections”), ions (see sheet “ions”), ions annotations (see sheet “annotation”) and intensities (see sheet “intensities”). References [71,72] are cited in the supplementary materials.
