# Supplementary material for: Metabolomics and Dual RNA-Sequencing on Root Nodules Revealed New Cellular Functions Controlled by Paraburkholderia phymatum NifA
Source: Metabolites. 2021 Jul 15;11(7):455. doi: 10.3390/metabo11070455 (PMC8305402; doi:10.3390/metabo11070455)
Supplement: Supplementary file 1 [file metabolites-11-00455-s001.zip › Table S7.pdf]

Table S7: Bacterial strains, plasmids and primers used in this work

Strain or plasmid	Description	Reference
Strains		
Escherichia coli		
Top10	Δ*lacX74 ara* Δ*139*Δ(*ara-leu*)	Invitrogen
Paraburkholderia phymatum		
STM815	Wild type	[1]
STM815-nifA$_{Pp}$	*nifA*$_{Pp}$::pSHAFT2 mutant of STM815; Cm^R	[2]
STM815-nifA$_{Pp}$-comp	STM815-nifA$_{Pp}$ mutant harboring pBBR1MCS-2-*nifA*; Km^R	This study
Plasmids		
pBBR1MCS-2	Broad host-range cloning vector; Km^R	[3]
pRK2013	Helper plasmid; Km^R	[4]
pBBR1MCS-2-*nifA*	pBBR1MCS-2 containing *nifA* (Bphy_7728) for complementation; Km^R	This study
**Primers**	**Sequence[1]**	**Source**
nifA_comp_F_HindIII	AAAAaagcttAATGCAAAAACGAGGCGTAG	This study
nifA_comp_R_XbaI	GGGGtctagaATGTACTCACGGGCCTTTTG	This study

[1]restriction sites are in lower letters.

References

1. Moulin, L.; Munive, A.; Dreyfus, B.; Boivin-Masson, C. Nodulation of legumes by members of the beta-subclass of Proteobacteria. *Nature* **2001**, *411*, 948, doi:https://doi.org/10.1038/35082070.

2. Lardi, M.; Liu, Y.; Purtschert, G.; de Campos, S.B.; Pessi, G. Transcriptome analysis of *Paraburkholderia phymatum* under nitrogen starvation and during symbiosis with *Phaseolus vulgaris*. *Genes (Basel).* **2017**, *8*, doi:10.3390/genes8120389.

3. Elzer, P.H.; Roop, R.M.; Kovach, M.E.; Robertson, G.T.; Peterson, K.M.; Steven Hill, D.; Farris, M.A. Four new derivatives of the broad-host-range cloning vector pBBR1MCS, carrying different antibiotic-resistance cassettes. *Gene* **1995**, *166*, 175–176, doi:10.1016/0378-1119(95)00584-1.

4. Phadnis, S.H.; Berg, D.E. Identification of base pairs in the outside end of insertion sequence IS*50* that are needed for IS*50* and Tn*5* transposition. *Proc. Natl. Acad. Sci. USA.* **1987**, *84*, 9118–9122, doi:10.1073/pnas.84.24.9118.
