# Supplementary figures and images for: Metabolomics and Dual RNA-Sequencing on Root Nodules Revealed New Cellular Functions Controlled by Paraburkholderia phymatum NifA
Source: Metabolites. 2021 Jul 15;11(7):455. doi: 10.3390/metabo11070455 (PMC8305402; doi:10.3390/metabo11070455)

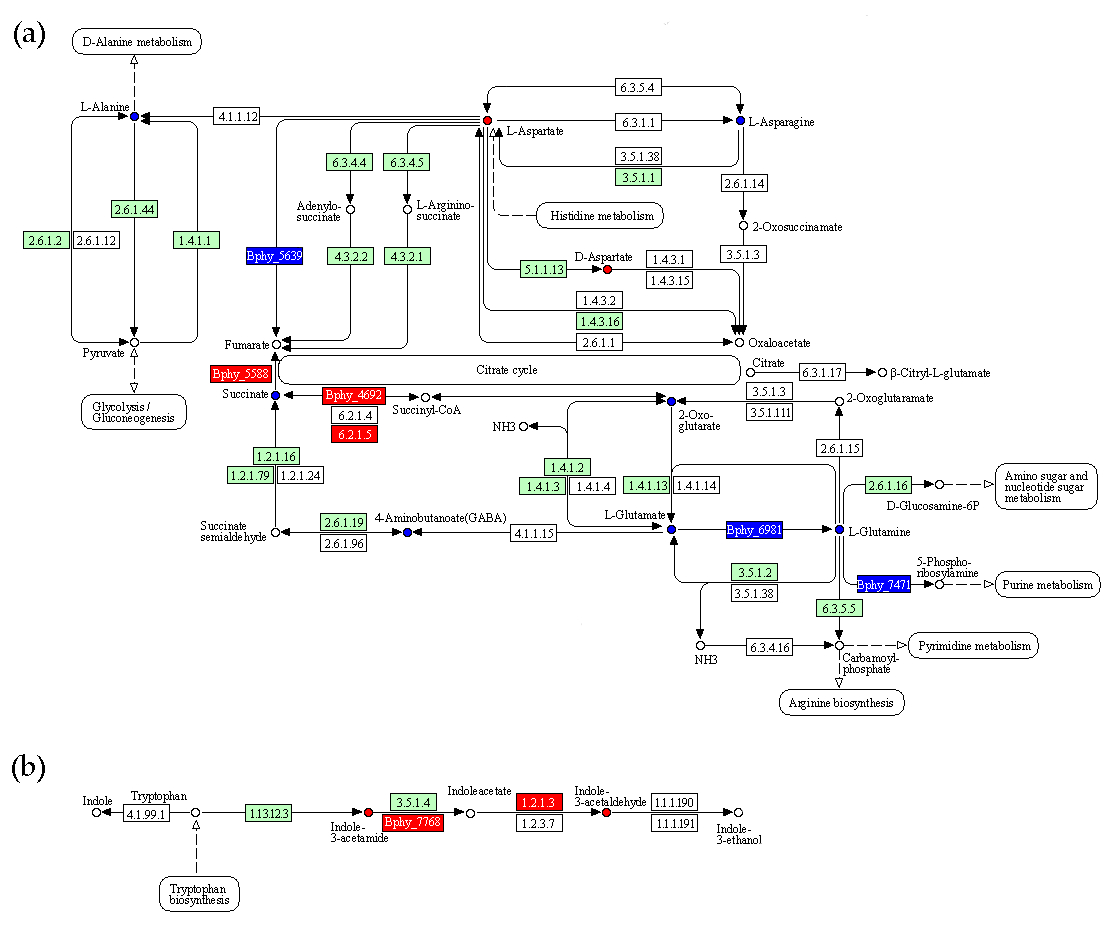

Supplement: Supplementary file 1 [file metabolites-11-00455-s001.zip › Figure S1.jpg]

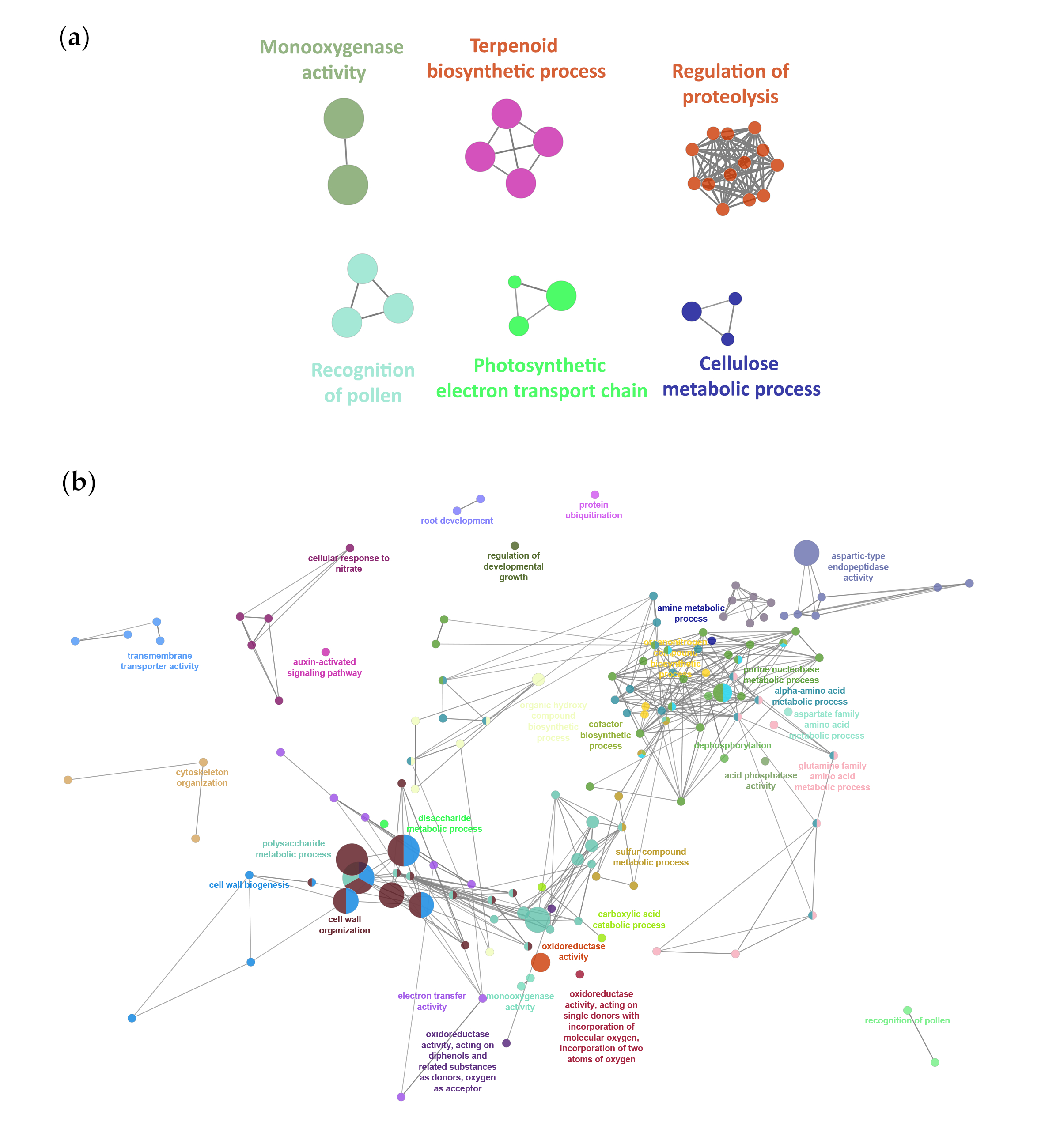

Supplement: Supplementary file 1 [file metabolites-11-00455-s001.zip › Figure S2.jpg]

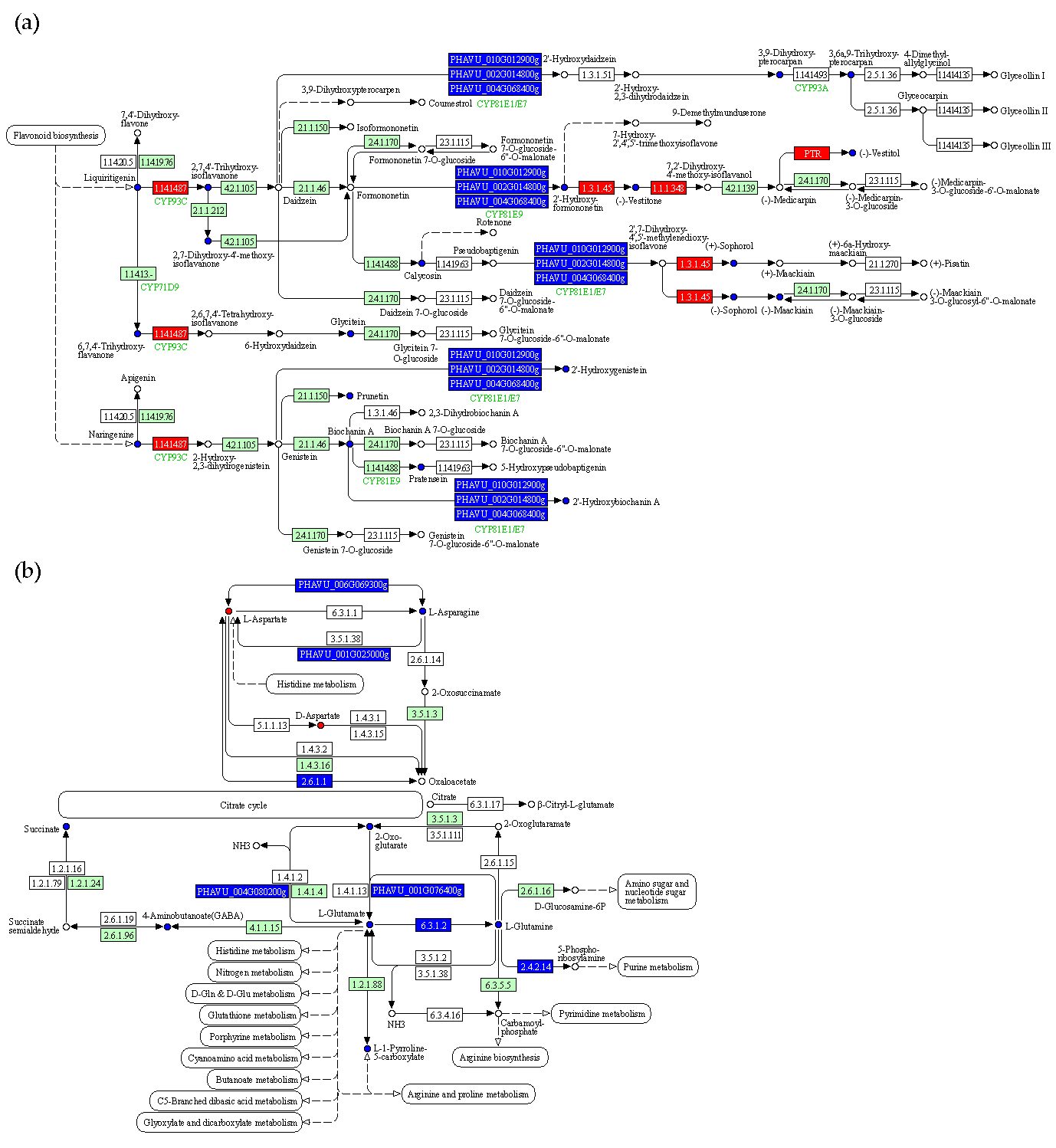

Supplement: Supplementary file 1 [file metabolites-11-00455-s001.zip › Figure S3.jpg]
